# Parallel evolution of linezolid-resistant *Staphylococcus aureus* in patients with cystic fibrosis

**DOI:** 10.1128/spectrum.02084-23

**Published:** 2023-09-19

**Authors:** Nicholas J. Pitcher, Andries Feder, Nicholas Bolden, Christian F. Zirbes, Anthony J. Pamatmat, Linda Boyken, Jared J. Hill, Alyssa R. Bartels, Andrew L. Thurman, Valerie C. Reeb, Harry S. Porterfield, Ahmed M. Moustafa, Paul J. Planet, Anthony J. Fischer

**Affiliations:** 1 Stead Family Department of Pediatrics, University of Iowa Carver College of Medicine, Iowa City, Iowa, USA; 2 Children’s Hospital of Philadelphia, Philadelphia, Pennsylvania, USA; 3 Perelman School of Medicine, University of Pennsylvania, Philadelphia, Pennsylvania, USA; 4 Department of Pathology, University of Iowa Carver College of Medicine, Iowa City, lowa, USA; 5 Department of Internal Medicine, University of Iowa Carver College of Medicine, Iowa City, lowa, USA; 6 State Hygienic Laboratory at the University of Iowa, Coralville, lowa, USA; 7 NIH Clinical Center, Bethesda, Maryland, USA; 8 Institute for Comparative Genomics, American Museum of Natural History, New York, New York, USA; University of Pittsburgh, Pittsburgh, Pennsylvania, USA

**Keywords:** *Staphylococcus aureus*, linezolid, MRSA, ribosomal RNA, hypermutation

## Abstract

Linezolid is an antibiotic used to treat serious *Staphylococcus aureus* infections. Resistance to linezolid is considered rare but could emerge with repeated dosing. We recently reported widespread prescription of linezolid for a cohort of patients with cystic fibrosis (CF). The goals of this study were to determine the incidence of linezolid-resistant methicillin-resistant *Staphylococcus aureus* (MRSA) in CF and identify molecular mechanisms for linezolid resistance. We identified patients who cultured *S. aureus* resistant to linezolid with minimum inhibitory concentration (MIC) >4 at the University of Iowa CF Center between 2008 and 2018. We obtained isolates from these patients and retested susceptibility to linezolid using broth microdilution. We used whole genome sequencing to perform phylogenetic analysis of linezolid-resistant isolates and examine sequences for mutations or accessory genes that confer linezolid resistance. Between 2008 and 2018, 111 patients received linezolid, and 4 of these patients cultured linezolid-resistant *S. aureus*. We sequenced 11 resistant and 21 susceptible isolates from these 4 subjects. Phylogenetic analysis indicated that linezolid resistance developed in ST5 or ST105 backgrounds. Three individuals had linezolid-resistant *S. aureus* with a G2576T mutation in 23S rRNA. One of these subjects additionally had a *mutS^−^ mutL^−^* hypermutating *S. aureus* that produced five resistant isolates with multiple ribosomal subunit mutations. In one subject, the genetic basis for linezolid resistance was unclear. We conclude that linezolid resistant *S. aureus* can occur through multiple genetic mechanisms in patients with repeated exposure to this antibiotic.

**IMPORTANCE:**

Patients with cystic fibrosis have persistent lung infections with *Staphylococcus aureus* that require extensive antibiotic treatments. Linezolid, an antibiotic given by oral or intravenous route, is prescribed repeatedly for patients whose lung disease has progressed. After treatment with linezolid, *S. aureus* strains can evolve antibiotic resistance through multiple genetic mechanisms. In addition to a common mutation in the 23S ribosomal RNA known to confer linezolid resistance, *S. aureus* strains can evolve novel resistance based on a combination of mutations affecting the bacterial ribosome. This combination of mutations was observed in a strain that exhibited hypermutation owing to the loss of the DNA repair genes *mutS* and *mutL*. In this cohort of patients with cystic fibrosis, linezolid resistance was transient, possibly due to the growth disadvantage of resistant strains. However, ongoing chronic exposure to linezolid may create optimal conditions for the future emergence of resistance to this critical antibiotic.

## INTRODUCTION

*Staphylococcus aureus* is the most prevalent pathogen affecting patients with cystic fibrosis (CF) in the United States (1). *S. aureus* infections are associated with increased airway inflammation in children with CF and precede worsening lung function (2, 3). Infections with *S. aureus* are difficult to eliminate, even with inhaled antibiotics (4). These durable infections persist indefinitely within patients, even after patients acquire *Pseudomonas aeruginosa* infections (5).

Methicillin-resistant *Staphylococcus aureus* (MRSA) infections are especially persistent in patients with CF (5). Because MRSA infections are associated with clinical worsening (3, 6), patients with MRSA receive intensified care, including frequent courses of antibiotics (7). Orally bioavailable antibiotics for MRSA include trimethoprim-sulfamethoxazole, tetracyclines, and oxazolidinones. Linezolid is an oxazolidinone antibiotic that is clinically effective in the treatment of severe *Staphylococcus aureus* respiratory infections (8), and it is commonly used in children and adults with CF (9).

Linezolid inhibits ribosomal protein synthesis and is broadly active against Gram-positive bacteria. It binds with high affinity to the ribosomal peptide-transferase center on the 50S subunit. This activity affects tRNA positioning and prohibits protein synthesis (10 – 12). Although resistance to linezolid is rare (13, 14), clinical linezolid-resistant bacteria have been reported shortly after the drug was introduced (15). Some linezolid-resistant bacteria carry mutations in domain V of the 23S rRNA gene, the most common of which is a G to T transversion at position 2576 according to the *Escherichia coli* numbering system (16). *Staphylococcus aureus* encodes multiple copies of the 23S rRNA gene. Thus, there is a gene dosage effect whereby the linezolid MIC increases with the number of mutant copies (17). Resistance to linezolid can also be conferred by the acquisition of *cfr*, which encodes a methyltransferase that methylates the 23S rRNA and is often carried on mobile genetic elements. In the absence of *cfr* or 23S rRNA mutations, resistance to macrolide, lincosamide, streptogramin, ketolide, and oxazolidinone (MLSKO) antibiotics has been reported in association with mutations of 50S ribosomal proteins or modifiers such as ribosomal proteins L4 and L22 (18).

Because MRSA is prevalent in patients with CF in the US, repeated prescription of linezolid for chronic, unremitting infections creates ideal conditions for evolving linezolid resistance. Additionally, as MRSA is a pathogen of global concern, it is increasingly important to understand how this organism develops resistance to critical antibiotics like linezolid. In this study, we aimed to determine the incidence of linezolid resistance in *S. aureus* from patients with CF and determine the molecular mechanisms of resistance in these strains.

## MATERIALS AND METHODS

### Subject selection and clinical information

All subjects were patients with CF who received treatment at the University of Iowa CF Center between 2008 and 2018. The diagnosis of CF was established by a positive sweat chloride test or genetic testing with two pathogenic mutations in *CFTR*. We excluded any subjects who did not have an electronic prescription or did not have a clinical microbiology report during the observation period. The primary exposure of interest was receiving an electronic prescription or inpatient order for linezolid. Electronic prescriptions became the standard of care within this center after February 2009. Linezolid exposure was defined by an electronic order for linezolid by either oral or intravenous (IV) route between February 2009 and April 2018. We recorded the indication for linezolid from each electronic prescription to determine whether it was intended for the treatment of *S. aureus* or for other CF pathogens such as non-tuberculous mycobacteria. We compared the prescription dates for linezolid versus other anti-staphylococcal medications to determine which medication(s) were used to treat infection and in what sequence. Clinical information about these patients, including lung function testing and other outcomes, was recorded to determine the severity of lung disease and the rate of complications. We analyzed clinical microbiology reports to determine positivity for relevant CF pathogens, including *S. aureus* and *P. aeruginosa*. The primary outcome of interest was the development of linezolid-resistant *S. aureus*.

### *S. aureus* isolates

The University of Iowa clinical microbiology laboratory routinely cryopreserved *S. aureus* isolates between 2009 and 2018. We requested any isolates that were cultured from patients who had at least one reported linezolid-resistant *S. aureus* on a clinical microbiology report. We confirmed linezolid resistance by microdilution antimicrobial susceptibility testing in Mueller-Hinton broth. We selected every isolate from individuals with at least one confirmed linezolid-resistant *S. aureus* culture, defined as MIC >4 µg/mL by broth microdilution, then analyzed isolates by whole genome sequencing (WGS). Of the 111 patients screened, we confirmed that 4 subjects had at least one linezolid-resistant *S. aureus*, including 11 linezolid-resistant isolates. A total of 32 isolates, 11 resistant and 21 susceptible, were available for analysis by WGS. Subject 1 had 6 resistant and 3 susceptible isolates; subject 2 had 1 resistant and 4 susceptible; subject 3 had 3 resistant and 2 susceptible; subject 4 had 1 resistant and 12 susceptible.

### Whole genome sequencing

To determine whether there was transmission of linezolid-resistant *S. aureus* between patients, and to determine the molecular mechanism for resistance, all isolates were analyzed by short-read WGS as previously described (19). DNA was isolated robotically at the University of Iowa State Hygienic Laboratory. We used Illumina DNA Prep and IDT for Illumina DNA/RNA UD indexes to prepare libraries and performed 2× 300 bp paired-end sequencing using the Illumina MiSeq Reagent v.3Kit.

### Long-read sequencing

To produce closed assemblies and resolve the number of rRNA operons per strain, we performed long-read sequencing. We selected six isolates (AF4001, AF4004, AF4005, HP20814.006, HP20814.043, and HP20814.062), providing one linezolid-resistant isolate per patient. We used the Rapid Barcoding Kit (SQK-RBK004) and flow cell type FLO-MIN106 on a MinION Mk1C device (Oxford Nanopore) using software v.21.11.6. The guppy component ont-guppy-for-mkac v.5.1.12 was used for base calling.

We created a hybrid assembly with short- and long-read sequences using Unicycler v.0.5.0 (20). To visualize 23S rRNA alignment, we used MegAlign Pro (DNASTAR) and *Escherichia coli* strain ATCC 8739 (GenBank accession no. NC_010468) to indicate position G2576 (21). We used DFast v.1.2.0 for web-based annotation of genomes after hybrid assembly to identify the location of transposable elements (22). Hybrid assemblies of AF4001 (subject 1), AF4005 (subject 2), AF4004 (subject 3), and HP20814.062 (subject 4) were used for reference-based assembly of any remaining isolates belonging to the same strain. To elucidate the genetic basis for linezolid resistance in AF4005, we used Snippy v.4.6.0 (23) to identify single nucleotide polymorphisms (SNPs) that were shared between HP20814.058, AF2246, AF2247, AF2248, and AF4007 compared to the hybrid assembly of AF4005 used as the reference genome.

### Phylogenetic analysis

Our phylogenetic analysis was completed using short-read sequences. We constructed a maximum likelihood tree for 68 genomes: 32 genomes from our collection and 36 assembled genomes available on GenBank (24), which were the top five genomes found using the topgenome (-t) feature of WhatsGNU (25). We processed the genomes from our collection using Bactopia v1.6.1 (26), and *de novo* assembly was completed using Shovill v1.1.0 (27). Sequence types were re-analyzed using mlst v2.19.0 (28), which made use of the PubMLST typing schemes (29). We determined the clonal complex for each genome using the WhatsGNU report. ABRicate (30) was used to determine the presence of *mecA* using a database containing genes from TCH1516 (GenBank Assembly no. GCF_000017095.1).

We annotated genomes using Prokka v1.14.6 (31). To infer an initial phylogenetic tree, we used the pangenome alignment produced by Roary v3.13.0 (32). We analyzed the core genome SNPs from the Roary output to produce a maximum likelihood phylogenetic tree in RAxML v8.2.9 (33). We used a GTR substitution model (34) to account for among-site rate heterogeneity using the Γ distribution and four rate categories (GTRGAMMA model) (35) for 100 individual searches with maximum parsimony random-addition starting trees. Node support was evaluated by running 100 non-parametric bootstrap pseudoreplicates (36). To calculate pairwise SNP distances, we used Roary to repeat the alignment of all isolates within each strain and the nearest strains from public databases. We produced an SNP distance matrix from the Roary core genome output using SNP-dist v0.8.2 (37). Intrastrain averages were calculated as the mean of all non-redundant pairwise distances between isolates within the strain.

To optimize visualization, we edited the phylogenetic tree using the iTol website (v6.4.2) (38). The data used in this publication were collected through the MENDEL high-performance computing (HPC) cluster at the American Museum of Natural History. This HPC cluster was developed with National Science Foundation Campus Cyberinfrastructure support through Award #1925590.

### Growth comparison

To compare the growth of linezolid-susceptible versus linezolid-resistant isolates, we inoculated 150 µL of broth containing isolates at an OD_600_ of 0.2 into 5 mL of tryptic soy broth in round bottom tubes. The tubes were incubated with shaking in a 37°C incubator. We assessed growth using an OD_600_ at 0, 2, 4, and 6 h. We recorded the results for the linezolid-resistant and -susceptible isolates from the same patients. The growth comparison was repeated on three different days; results from a representative experiment are shown. For statistical testing, we used a two-way ANOVA with pairwise comparisons at each time point.

### Statistical methods

To compare categorical variables for subjects who were prescribed linezolid versus those who were not, we used Fisher’s exact test. To compare ages, we used the Wilcoxon rank sum test to compare birth years as a continuous variable. We used RStudio v.2022.12.0+353 or GraphPad Prism v.9 for statistical tests.

## RESULTS

### Prescription of linezolid in cystic fibrosis

We surveyed electronic prescriptions and inpatient orders to identify patients receiving linezolid. Three hundred sixty patients with CF (with or without a lung transplant) had encounters between 2009 and 2018, of whom 346 had both medication information and microbiology results. Of these patients, 111 (32%) were treated with linezolid (Fig. S2 and S3). We found 491 oral prescriptions and 343 intravenous prescriptions, consistent with mixed inpatient and outpatient uses of linezolid.

### Patient characteristics

We compared patients receiving linezolid to control patients who received other antibiotics. Although it treats other pathogens such as non-tuberculous mycobacteria (39), linezolid treatment was strongly associated with MRSA and other antibiotics targeting MRSA (Table 1). MRSA infections are associated with increased age and poorer outcomes in CF (3, 6, 40). We found that patients prescribed linezolid were older and more likely to have died or required a lung transplant than controls. These patients were also more likely to have coexisting infections with *P. aeruginosa* and other CF-associated Gram-negative pathogens that are common with advanced lung disease. The increased disease progression likely increased their risk of receiving repeated courses of antibiotics, alone or in combination.

**TABLE 1:** Clinical characteristics of patients prescribed linezolid therapy from 2009 to 2018

Characteristic	Linezolid prescribed	Linezolid not prescribed	*P*
**Number**	111	235	
**Sex—female**	56 (50.5%)	109 (46.4%)	0.49
***CFTR*** **genotype**			0.08, df = 2
F508del/F508del	66 (59.5%)	112 (47.7%)	
F508del/other	34 (30.6%)	101 (43.0%)	
Other/other	11 (9.9%)	22 (9.4%)	
**CFTR modulator therapy**			
Ivacaftor	6 (5.4%)	23 (9.8%)	0.21
Ivacaftor, lumacaftor/ivacaftor, or tezacaftor/ivacaftor	40 (36.0%)	81 (34.5%)	0.81
**Anti-staphylococcal antibiotics**			
Trimethoprim-sulfamethoxazole	93 (83.8%)	138 (58.7%)	<0.001
Tetracycline, doxycycline, or minocycline	68 (61.3%)	52 (22.1%)	<0.001
Vancomycin IV	96 (86.5%)	47 (20.0%)	<0.001
Vancomycin inhaled	14 (12.6%)	0	<0.001
**Birth year**, median (IQR)	1992 (1982–2000)	1997 (1983–2009)	0.002^*b*^
**Outcome, as of 31 Dec. 2018** ^***a***^			<0.001, df = 4
Death without lung transplant	10 (9.0%)	14 (6.0%)	
Death following lung transplant	14 (12.6%)	4 (1.7%)	
Alive with lung transplant	14 (12.6%)	4 (1.7%)	
Alive without lung transplant, culture after 1 Jan. 2017	62 (55.9%)	170 (72.3%)	
No known death or transplant, data missing after 1 Jan. 2017	11 (9.9%)	43 (18.3%)	
**Pathogen positivity, 2008–2018**			
MRSA	94 (84.7%)	58 (24.7%)	<0.001
Methicillin susceptible *S. aureus* (MSSA)	79 (71.2%)	170 (72.3%)	0.90
*P. aeruginosa*	103 (92.8%)	160 (68.1%)	<0.001
Mucoid *P. aeruginosa*	77 (69.4%)	92 (39.1%)	<0.001
*Burkholderia* spp.	13 (11.2%)	13 (5.5%)	0.05
*Stenotrophomonas maltophilia*	58 (52.3%)	90 (38.3%)	0.02
*Achromobacter xylosoxidans*	18 (16.2%)	16 (6.8%)	0.01

^*a*^ *CFTR* genotypes and the categorical outcomes reported are mutually exclusive. *P* values for categorical outcomes are calculated using Fisher’s exact test. The degrees of freedom (df) for each test = 1 unless otherwise indicated.

^*b*^ For birth year, the *P* value is determined by the Wilcoxon rank sum test.

### Linezolid prescriptions are given to treat *S. aureus* infections and followed other attempts to treat MRSA

The indication for linezolid was written for 197 (40%) of the oral prescriptions. The most common indication for linezolid was the treatment of *S. aureus* or MRSA (*N* = 131), followed by treatment of CF, bronchitis, or pulmonary exacerbation (*N* = 57), followed by non-tuberculous mycobacteria (*N* = 4).

Three other antibiotic classes are commonly used to treat MRSA in CF: vancomycin, trimethoprim-sulfamethoxazole, and tetracyclines (40). In our cohort, 53 patients were treated with all four classes. Within these patients, trimethoprim/sulfamethoxazole was used first, followed by vancomycin, then linezolid, and tetracyclines (Fig. S4). These data suggest that linezolid was usually reserved for later attempts at treating MRSA.

### Repeated prescription of linezolid

Repeated linezolid dosing in patients with chronic infections may increase the risk of evolving resistance. Patients who were prescribed linezolid received repeated prescriptions of linezolid and other antibiotics with activity against MRSA (Fig. S5). Of the 111 subjects receiving linezolid, 27 received only 1 prescription, whereas 62 patients received 5 or more prescriptions (Fig. S6). The maximum number of linezolid prescriptions written for a single patient was 50. Linezolid dosing was usually prescribed at the typical adult dose of 600 mg twice daily for 14 days, but several patients received prescriptions indicating one or more refills (Fig. S7, S8, and S9).

### Resistance of *S. aureus* to linezolid

**FIG 1:**
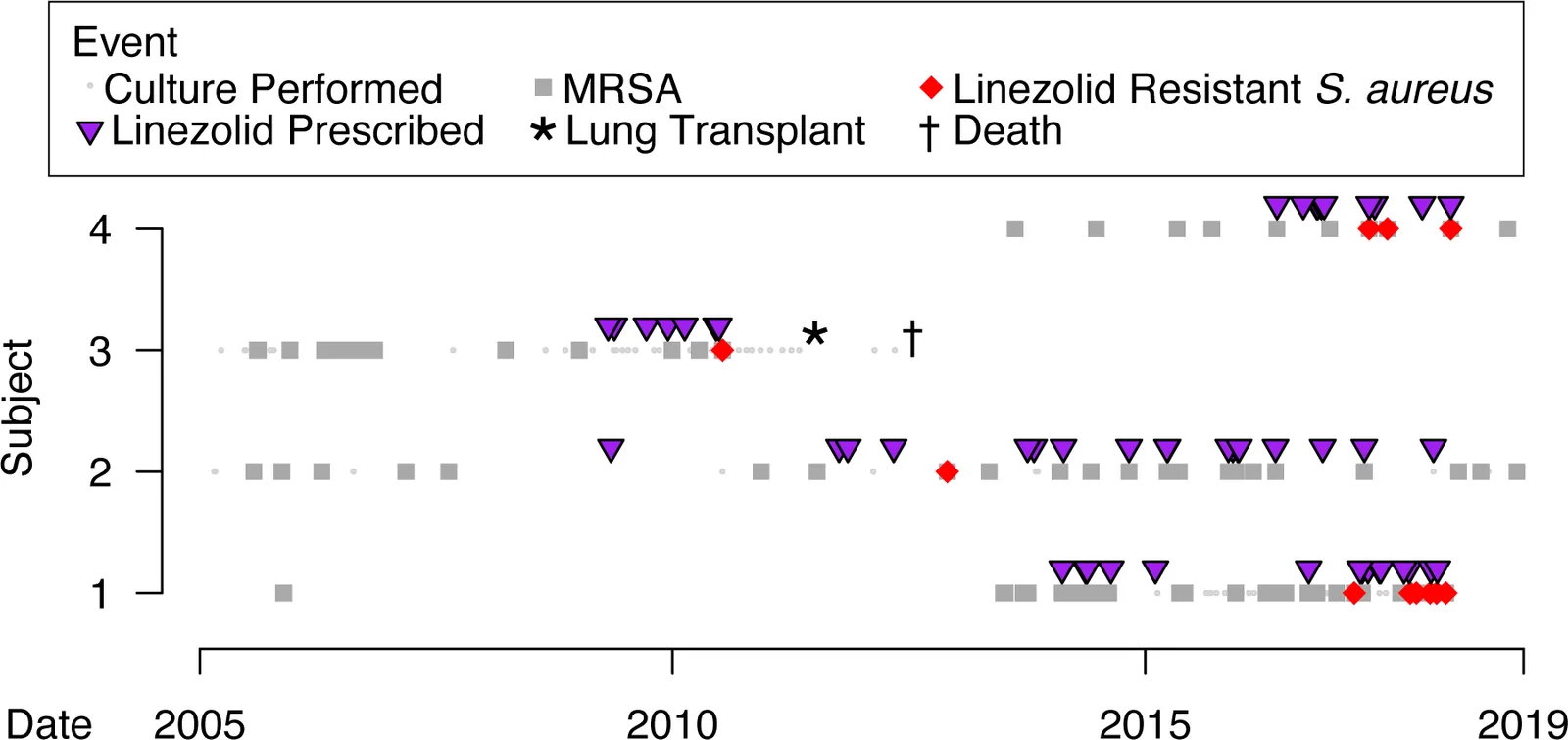
Timelines of *S. aureus* positivity and linezolid prescription in four patients with cystic fibrosis who had confirmed linezolid-resistant MRSA. Small dots indicate that a culture was performed. Dark gray squares indicate MRSA. Red diamonds indicate linezolid-resistant *S. aureus*. Purple triangles indicate linezolid prescriptions.

Linezolid-resistant *S. aureus* were reported in 11 patient encounters for 5 patients. We obtained *S. aureus* isolates, including linezolid-susceptible and -resistant strains, from these patients and performed antibiotic susceptibility testing by broth microdilution. We confirmed linezolid resistance (MIC ≥8 mg/L) in isolates from 4 of the 5 subjects. These 4 patients received between 8 and 20 orders of linezolid (Fig. 1). Each of these four subjects received a linezolid prescription allowing at least one refill. Linezolid-resistant *S. aureus* appeared only after patients received linezolid. Each subject had at least one susceptible isolate after linezolid exposure. We analyzed 11 linezolid-resistant and 21 linezolid-susceptible isolates by WGS (Table 2).

**TABLE 2:** Isolates analyzed by WGS

Subject, first linezolid year-month	Isolate	Collection year-month	Linezolid MIC	Oxacillin MIC	23S rRNA copy number^*a*^	MLST^*b*^	Mechanism of resistance
Subject #1, 2014-02	AF4001	2015-06	4	16	6	5	
	HP20814.006	2017-01	4	>16	6	5	
	AF2324	2018-01	>8	>16		5	G2576T
	HP20814.043	2017-03	8	>16	6	ND	Multiple
	HP20814.051	2017-04	4	>16		ND	
	AF4003	2017-10	8	>16		ND	Multiple
	HP20814.091	2017-11	8	>16		ND	Multiple
	AF2323	2018-01	8	>16		ND	Multiple
	AF2325	2018-03	8	>16		ND	Multiple
Subject #2, 2009-05	AF4005	2012-11	8	>16	5	105	Unknown
	HP20814.058	2017-04	4	>16		105	
	AF2246	2018-04	4	>16		105	
	AF2247	2018-07	2	>16		105	
	AF2248	2018-12	2	>16		105	
Subject #3, 2009-04	AF4006	2006-04	4	>16		5	
	AF4008	2006-06	4	>16		5	
	AF4009	2006-06	4	>16		5	
	AF4010	2006-07	4	>16		5	
	AF4011	2006-08	2	>16		5	
	AF4012	2006-09	2	>16		5	
	AF4013	2006-11	2	>16		5	
	AF4015	2009-01	4	>16		5	
	AF4016	2009-12	4	>16		5	
	AF4017	2010-04	4	>16		5	
	AF4004	2010-07	8	16	5	5	G2576T
	AF4007	2006-05	4	>16		105	
	AF4014	2008-03	4	>16		582	
Subject #4, 2016-05	AF1552	2015-09	4	0.25		5	
	HP20814.062	2017-05	8	>16	6	5	G2576T
	AF4002	2017-07	8	>16		5	G2576T
	AF2001	2018-03	>8	>16		5	G2576T
	AF2002	2018-11	4	4		5	

^*a*^ The number of rRNA copies is based on hybrid assembly using long- (Oxford Nanopore Minion) and short-read (Illumina MiSeq) sequences using Unicycler software version 0.5.0.

^*b*^ MLST entries listed as “ND” indicate that the sequence type was not determined owing to the absence of at least one gene.

### Whole genome sequencing analysis

All 11 linezolid-resistant isolates evolved from either ST5 or ST105 MRSA backgrounds. These clonal complex 5 lineages are usually SCC*mec* II positive and considered hospital-associated MRSA. Collectively, these sequence types are the most prevalent MRSA lineages in our center and are associated with lower lung function in younger patients compared to clonal complex 8 MRSA lineages (19).

A previous report suggested that these hospital-acquired MRSA lineages have greater potential to evolve resistance to protein synthesis inhibitor drugs due to a lower rRNA copy number (41). Determining the number of rRNA copies and localizing rRNA mutations to a single operon is challenging with short-read sequencing alone because the rRNA sequences are nearly identical at each locus. To determine rRNA copies, we used hybrid assembly with short- and long-read WGS. We selected six isolates for hybrid assembly, including one linezolid-resistant isolate from three patients and three isolates (one resistant and two susceptible) from a subject who cultured six linezolid-resistant isolates. Two subjects had MRSA with six rRNA operons; the other two had MRSA with five rRNA operons (Table 2). Thus, linezolid resistance was not limited to strains with a lower rRNA copy number.

### Assignment of isolates into strains by clade breaker

**FIG 2:**
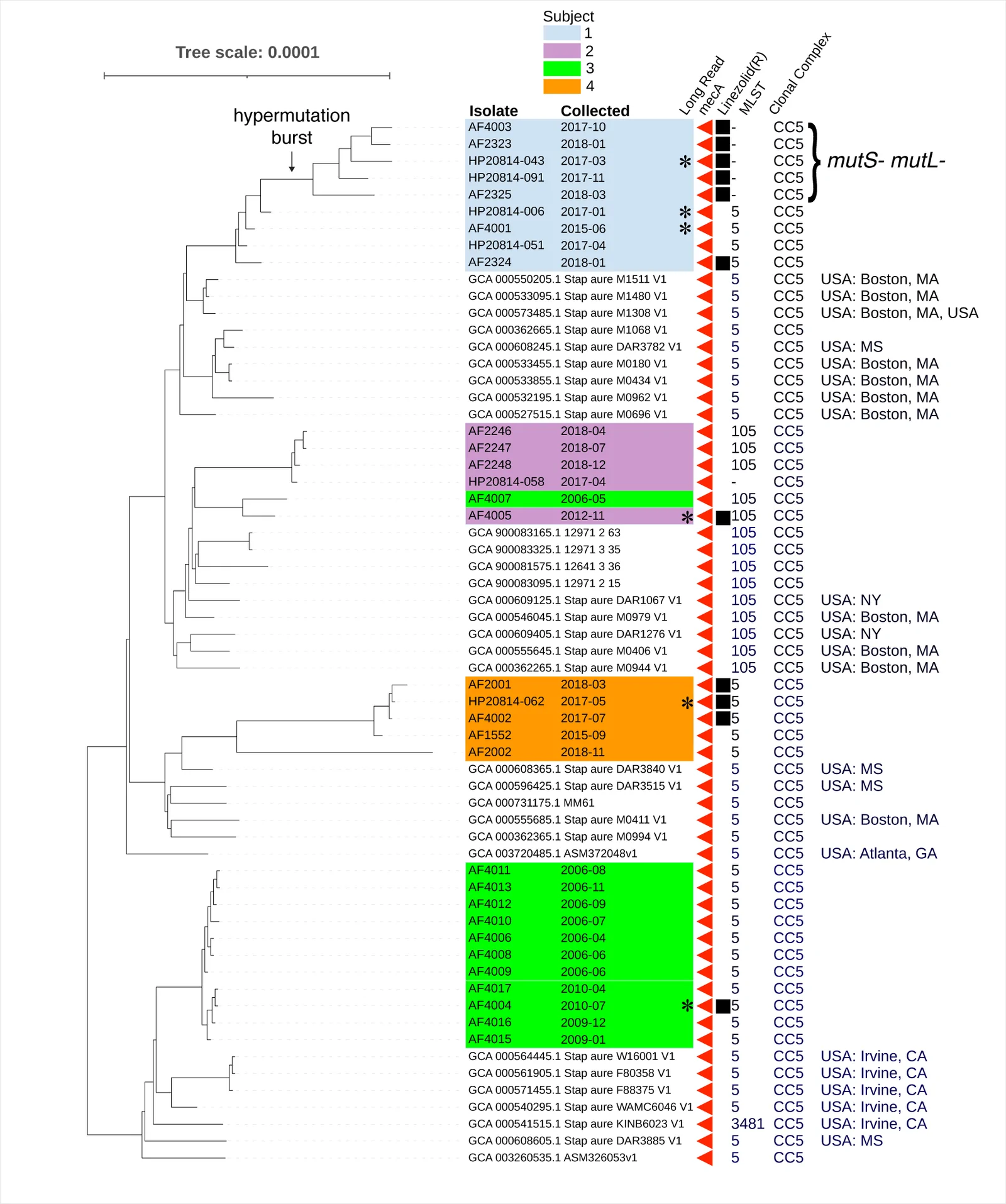
Maximum likelihood phylogenetic tree containing clonal complex 5 linezolid-resistant *S. aureus* isolates, presented in the context of closely related *S. aureus* genomes available in public databases. Linezolid-resistant isolates (MIC >4) are indicated by a black square, and MRSA isolates are indicated by a red triangle. The collection times (year and month) are indicated for each isolate. Isolates presented in this study are color-coded by study subject, whereas non-colored branches represent genomes identified from GenBank that were among the top five most closely related to genomes from our study. The geographic origins of genomes found in GenBank are given in the right margin. Branch lengths are drawn proportionate to nucleotide substitutions per site. This phylogenetic tree omits one isolate, AF4014, that did not belong to clonal complex 5. A phylogenetic tree including this isolate is provided in Fig. S10. The confidence level for each node of the phylogenetic tree is provided in Fig. S11. Five linezolid-resistant isolates from subject 1 (AF2325, HP20814-091, HP20814-043, AF2323, and AF4003) had complete loss of *mutS* and *mutL* associated with hypermutation, as shown by the increased branch length indicated by the arrow. Similarly, AF2002 from subject 4 had an increased number of substitutions; this isolate had a p.P340fs mutation from the loss of an adenine within a 9-adenine stretch. Isolates with further analysis by long-read sequencing are indicated with an asterisk. These selected isolates included at least one linezolid-resistant *S. aureus* from each subject and two susceptible isolates from subject 1 that immediately preceded the hypermutation burst.

Determining whether linezolid-resistant MRSA spreads between patients requires a working definition of a strain. For acute outbreaks related to the spread of MRSA isolates taken from different patients, each isolate may be highly similar (SNP distance <20) (42). In chronic infections like CF, there can be greater diversity within a single patient. Therefore, the first step in analyzing these isolates is to assign them to strains. Some patients with CF are infected by multiple strains of *S. aureus*, and occasionally unrelated patients share the same strain (39). To determine whether there was sharing of linezolid-resistant strains between patients, we performed phylogenetic analysis to assign closely related isolates to strains. Because some CF-associated MRSA strains exhibit hypermutation (19) or have increased SNP distance as they develop linezolid resistance (43, 44), we did not use strict SNP cutoffs to divide isolates into strains. Instead, we used a clade-breaker approach (45). For each isolate, we downloaded the five most closely related *S. aureus* genomes from GenBank. If a given isolate from the patient was more closely related to an independent strain from GenBank than it was to another isolate taken from the same patient, we assumed that the patient’s infection was polyclonal, as the patient’s two isolates were separated by a “clade-breaker.” Results of the phylogenetic analysis are shown in Fig. 2; Fig. S10 and S11. The clade breaker method helped classify several isolates that would have had ambiguous strain assignments using a strict SNP distance relationship alone; several pairwise SNP distances within strains were >60, and some of the pairwise SNP distances within strains approached the pairwise distances measured between distinct strains isolated from different subjects, which were generally >200 (Fig. S12 to S16).

### Linezolid resistance associated with 23S rRNA mutations evolved independently in multiple strains

We previously reported that closely related MRSA was occasionally shared between patients (19). Sharing of strains would suggest transmission of resistant organisms between patients. If this were to occur with linezolid resistance, it would threaten linezolid effectiveness across the CF population.

Linezolid-resistant *S. aureus* isolates, labeled in Fig. 2 with black squares, appeared in five separate clusters. Within subject 1, linezolid resistance evolved twice from the patient’s ST5 ancestor. One of the linezolid-resistant isolates (AF2324) diverged from the earliest common ancestor, whereas a second series of five linezolid-resistant isolates diverged later. Long-read sequencing of AF2324 revealed six copies of the 23S rRNA operon and confirmed two copies of a G2576T mutation known to cause linezolid resistance (Fig. S17). The remaining five isolates from this subject had normal 23S rRNA, implying an independent resistance mechanism.

### Linezolid resistance on a hypermutator strain background

We examined the remaining linezolid isolates from subject 1 further. Although they were descended from ST5, these isolates were not assigned a sequence type owing to disruption of the MLST gene *glpF*. Furthermore, short-read sequences did not detect the neighboring genes *mutS* and *mutL*, which play important roles in DNA mismatch repair. Drawing the phylogenetic tree with branch lengths proportional to nucleotide polymorphisms, the isolates lacking *mutS* and *mutL* had longer inferred branch lengths, suggesting a hypermutator phenotype (Fig. 2). To determine how these isolates lost *mutS* and *mutL*, we used long-read sequencing to compare isolates with *mutS* and *mutL* (AF4001 and HP20814-006) to an isolate from the same strain that was lacking these genes (HP20814-043). We deliberately selected AF4001 and HP20814-006 for the long-read sequencing comparison because of their positions on the phylogenetic tree, which indicated their close relationship with the ancestor of the series of isolates lacking *mutS* and *mutL*.

**FIG 3:**
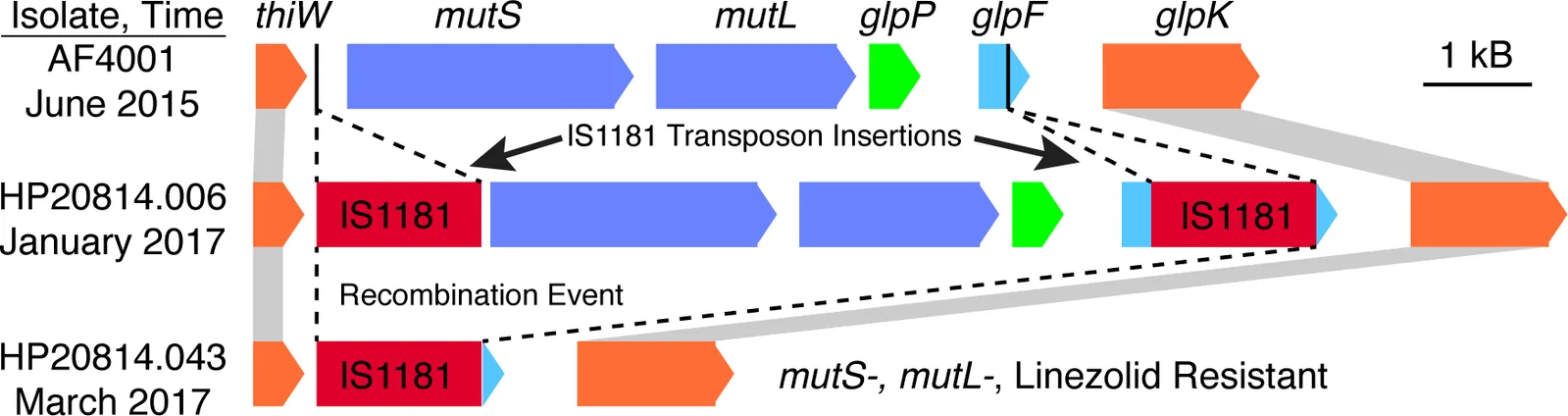
Genetic deletion of the DNA mismatch repair genes *mutS* and *mutL* in a strain from subject 1 that developed linezolid resistance. Isolates were taken at different time points. *S. aureus* chromosome assemblies from short- and long-read sequences are presented for genes near *mutS* and *mutL*, which are colored purple. In the second isolate, HP20814.006, transposon IS1181 (red) was inserted twice within the locus, including 5′ of *mutS* and within the coding sequence of *glpF*. A subsequent recombination event deleted *mutS*, *mutL*, *glpP*, and the 5′ coding sequence of *glpF*. In addition to HP20814.043, four other linezolid-resistant isolates lacked *mutS* and *mutL* in this patient. The undisturbed genes *thiW* and *glpK* that flank the region are indicated in orange and are connected by gray shading.

We created new hybrid assemblies using long- and short-read sequences for these three isolates. The addition of long-read sequencing helped identify that all three isolates had amplified copies of a transposable element, *IS1181*. Each isolate had at least 40 copies of *IS1181* distributed throughout the genome. *IS1181* was originally absent near *mutS* and *mutL* as shown for AF4001 (Fig. 3) but was subsequently inserted twice flanking these genes. As shown in HP20814-006, *IS1181* was inserted within *glpF* and upstream of *mutS*. A subsequent recombination event between these transposable elements resulted in the complete excision of *mutS*, *mutL*, *glpP*, and the 5′ portion of *glpF*.

Following the loss of *mutS* and *mutL*, all subsequent isolates were linezolid-resistant despite a normal 23S rRNA sequence. Instead, the strain accumulated a series of mutations in genes that encode ribosomal accessory proteins or modifiers (Table 3). These mutations included a previously reported variant p.S145del in *rplC* as well as new variants in the 50S ribosomal genes *rlmI*, *rluD*, and *rlmN*. Additionally, the strain accumulated mutations affecting the 30S ribosomal subunit, which could facilitate resistance to other protein synthesis inhibitors like tetracyclines.

**TABLE 3:** A combination of ribosomal variants in linezolid-resistant *S. aureus* arising from a mutator background

		DNA repair	50S ribosome proteins/modifiers							30S ribosome proteins/modifiers		
Isolate	Linezolid MIC	*mutS, mutL* SA_RS06425 SA_RS06430	23S rRNA G2576^*a*^	*cfr*	*rplC* SA_RS11765	*rlmI* SA_RS05285	*rluD* SA_RS05915	*rlmN* SA_RS06020	*rplD* SA_RS11760	*rpsC* SA_RS11735	*rpsJ* SA_RS11770	*rpsD* SA_RS08675
HP20814.051	4	wt	wt	Absent	wt	wt	wt	wt	wt	wt	wt	wt
AF4001	4	wt	wt	Absent	p.S145del	wt	wt	wt	wt	wt	wt	wt
HP20814.006	4	wt	wt	Absent	wt	wt	wt	wt	wt	wt	p.K57M	wt
AF2325	8	Deleted	wt	Absent	p.S145del	p.V218G	wt	wt	wt	wt	p.V55A, p.K57N, p.Y58C	wt
HP20814.091	8	Deleted	wt	Absent	p.S145del	p.V218G	p.L158F	wt	wt	wt	p.K57N, p.Y58C	p.L193S
HP20814.043	8	Deleted	wt	Absent	p.S145del	p.V218G	p.L158F	wt	wt	wt	p.K57N, p.Y58C	p.L193S
AF2323	8	Deleted	wt	Absent	p.S145del	p.V218G	p.L158F	p.Q52fs	wt	wt	p.K57N, p.Y58C	p.L193S
AF4003	8	Deleted	wt	Absent	p.S145del	p.V218G	p.L158F	p.Q52fs	wt	wt	p.K57N, p.Y58C	p.L193S
AF2324	>8	wt	G2576T	Absent	wt	wt	wt	wt	wt	wt	p.K57R	wt

^*a*^ 23S rRNA nomenclature is according to *Escherichia coli* strain ATCC 8739 (GenBank accession no. NC_010468). Reference sequences for each gene are indicated below the gene name. “wt” indicates the sequence is wild type.

### Sharing of a strain capable of linezolid resistance

As reported by others, we observed examples of polyclonality in *S. aureus* and shared strains even in this small patient sample (39). Subject 3 had a polyclonal infection, including three distinct *S. aureus* strains (Fig. 2). One isolate was ST105 (AF4007), belonging to the same strain infecting subject 2 (AF4005). AF4005 was linezolid-resistant, suggesting that direct or indirect transmission of a strain capable of evolving linezolid resistance occurred between two different patients. These isolates were cultured 6 years apart, and it is unclear how this strain was shared.

We attempted to determine the genetic basis for linezolid resistance in AF4005. Its genome did not encode *cfr*, lacked the G2576T transversion in 23S rRNA, and did not have mutations in ribosomal protein modifiers that we identified in the mutator strain from subject 1. We aligned the genomes of each of the linezolid-susceptible isolates from this strain (HP20814.058, AF2246, AF2247, AF2248, and AF4007) to the hybrid assembly for AF4005 to identify genome-wide SNPs that were unique to AF4005. Thirty-five SNPs met these criteria. The most plausible variant was a frameshift mutation in a GbsR/MarR family transcriptional regulator. Other possibilities included an SNP in a non-coding region downstream of *vraG*, an SNP upstream of the sigma factor *sigS*, and a p.V179I substitution in *fusA*. The molecular basis for linezolid resistance in this isolate remains unclear.

The strain that would ultimately evolve a linezolid-resistant isolate in subject 3 (AF4004) was independent of the strain shared with subject 2. AF4004 was ST5 and had G2576T mutations in the 23S rRNA. Thus, although there was strain sharing between subjects 2 and 3, the evolution of linezolid resistance was phylogenetically and mechanistically distinct in both patients.

Subject 4 had three linezolid-resistant isolates, all of which had G2576T mutations. An example from this strain is given in Fig. S17. These isolates belonged to the same strain and were independent of strains obtained from other subjects. Together, resistance to linezolid developed independently in each of the four subjects. G2576T mutations explained the linezolid resistance that appeared in three subjects. One subject had linezolid resistance that evolved at least twice during the observation period.

### Growth disadvantage of linezolid-resistant *S. aureus* isolates

**FIG 4:**
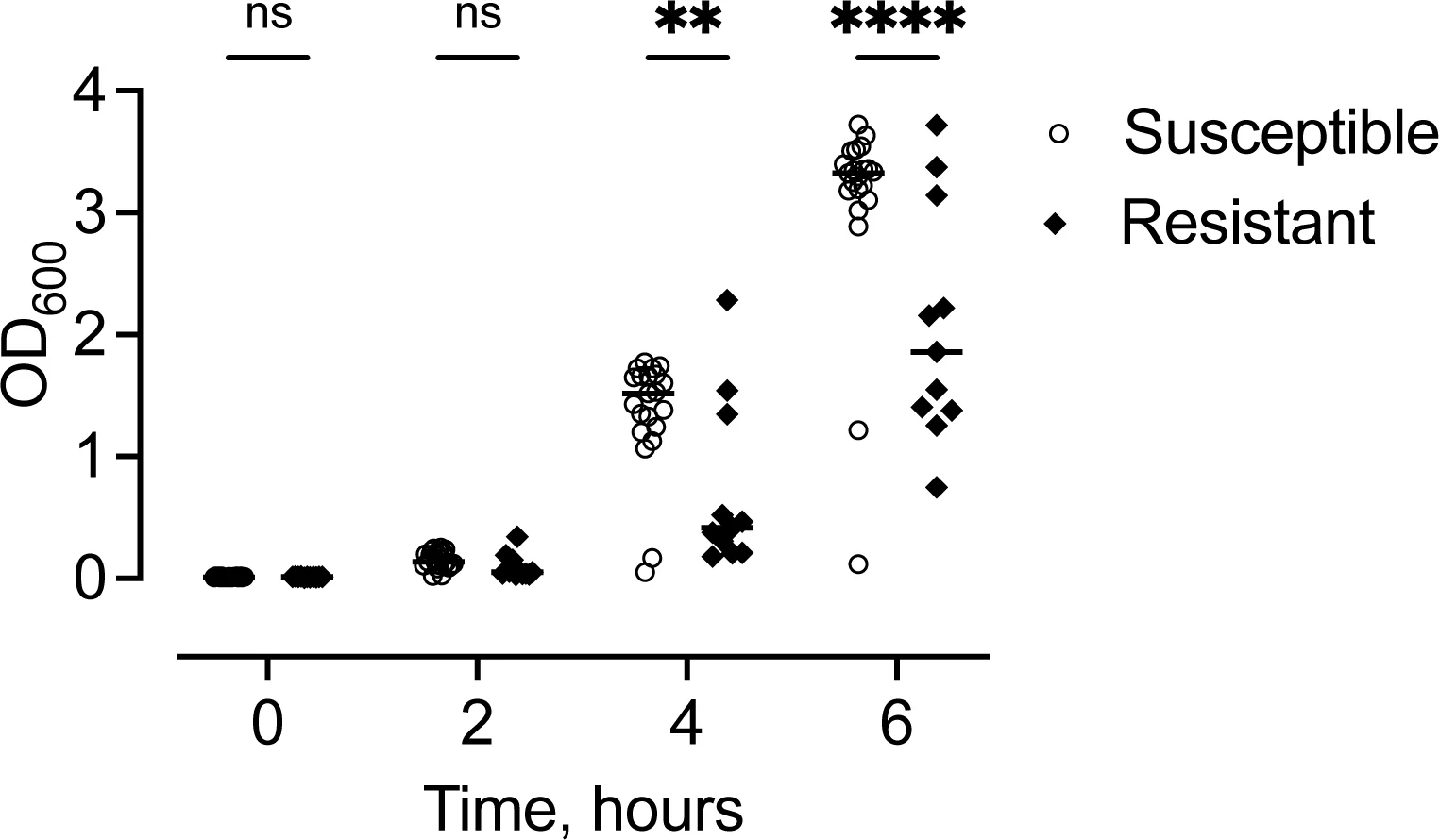
Growth of linezolid-susceptible and linezolid-resistant *S. aureus* from patients with CF in tryptic soy broth. On average, the growth of linezolid-resistant *S. aureus* is slower than that of linezolid-susceptible isolates. Two-way ANOVA with pairwise comparisons at each time point. Each dot represents a different isolate, and lines indicate the median. *N* = 21 susceptible and 11 resistant isolates; **, *P* < 0.01; ****, *P* < 0.0001; ns, not significant.

Although linezolid resistance should be an advantageous phenotype for *S. aureus*, we were surprised to see that some linezolid-resistant strains appeared only transiently despite continued antibiotic pressure. The lack of persistent linezolid-resistant *S. aureus* suggests that it is at a growth disadvantage compared to linezolid-susceptible *S. aureus* (46). Therefore, we compared the growth of linezolid-resistant *S. aureus* to linezolid-susceptible *S. aureus* isolated from the same patients (Fig. 4). On average, linezolid-susceptible isolates grew significantly better at 4 (*P* < 0.01) and 6 h (*P* < 0.0001) than linezolid-resistant isolates, confirming that linezolid resistance comes with a fitness cost, perhaps accounting for low persistence in the CF airway.

## DISCUSSION

We show that of the 111 patients treated with linezolid at the University of Iowa CF Center over nearly a decade, 4 developed confirmed linezolid-resistant *S. aureus*, an incidence of approximately 4 per 1,000 person-years. Subjects who received linezolid often received repeated antibiotic courses because of persistent MRSA. In general, these patients received linezolid after first taking other anti-staphylococcal treatments. These patients had lower lung function than patients who did not receive linezolid. When an indication was provided, linezolid was prescribed more often for treating persistent *S. aureus* infections than other CF pathogens like non-tuberculous mycobacteria.

The strains of linezolid-resistant *S. aureus* that we examined evolved independently in each patient without acquisition of *cfr*. The most common genetic explanation for linezolid resistance was a well-known G2576T mutation in the 23S rRNA. Notably, one subject evolved linezolid resistance twice by two independent mechanisms. In one strain from this subject, the loss of the DNA repair genes *mutS* and *mutL* facilitated a series of mutations in the 50S ribosomal subunit that may have exerted a combinatorial effect on linezolid resistance.

### Comparison to previous studies

The G2576T transversion in 23S rRNA is a frequently reported mechanism of linezolid resistance (15, 44, 47 – 50). Three patients in this study had *S. aureus* with this mutation, which evolved independently in each case. Mutations in the ribosomal L3 protein that contribute to linezolid resistance are rare. Five isolates in a mutator lineage contained a Δser145 variant previously reported in association with linezolid resistance (51). However, expression of this mutation in *E. coli* was not sufficient for linezolid resistance (51). Two of the isolates in this study contained frameshift mutations in the *rlmN* methyltransferase. While this mutation alone is typically not sufficient to confer linezolid resistance, LaMarre et al. showed that inactivation of RlmN results in increased linezolid MIC (52). It is possible that these mutations act synergistically with other mutations that alter the 50S ribosome to further adapt to linezolid.

Hypermutating bacterial strains can develop resistance to multiple antibiotics (53 – 55). Ba et al. observed that linezolid resistance develops in *Enterococcus faecalis* mutators, but resistance does not develop in laboratory strains of *S. aureus* with targeted disruption of DNA repair genes (56). It is possible that linezolid resistance did not occur under these settings because evolving resistance may require time to develop a combination of several point mutations. By contrast, antibiotic resistance increased following disruption of *mutL* or *mutS* by frameshift mutations in *P. aeruginosa* (19). Our study shows that linezolid-resistant *S. aureus* can evolve into a strain with genetic features of hypermutation, including the loss of DNA genes and an increased number of SNPs. The mechanism of hypermutation was related to the complete loss of *mutL* or *mutS* from a recombination event, which facilitated resistance to linezolid by a series of mutations affecting the 50S ribosome subunit. Insertional inactivation of DNA repair genes has been suggested to have a stronger predisposition to antibiotic resistance than potentially reversible point mutations (57).

### Advantages of our approach

Because we had nearly complete electronic prescription information in a stable patient cohort, we had a quantitative measure of prolonged exposure to linezolid and other anti-staphylococcal antibiotics in a population at risk of linezolid resistance. The combined use of short- and long-read WGS allowed us to determine the number of rRNA operons and visualize structural rearrangements in the *S. aureus* chromosome. With these resources, we were able to capture changes in the genome outside of the predicted antibiotic targets. Because some strains have hypermutation features, the use of a clade-breaker phylogenetic approach allowed flexibility in determining strain assignments despite occasional large differences in SNPs between isolates collected from the same subject. Longitudinal data collection, including linezolid-susceptible and -resistant isolates, allowed us to infer the order of mutations within individual patients and to assess how linezolid resistance evolved. While short-read sequencing was sufficient for constructing a phylogenetic tree and identifying common mutations like G2576T that confer linezolid resistance, we found that there was added value in long-read sequencing. This step provided better genome assembly through genes existing in multiple copies, including rRNA operons and insertional elements like IS1181. This ultimately suggested the genetic origin of hypermutation in a strain lacking *mutS* and *mutL*.

### Study limitations

Our study was single-centered, so our findings may not generalize to centers that prescribe less linezolid. Our approach could also underestimate the incidence of linezolid resistance. We studied single isolates per study encounter. Because we found that linezolid-resistant *S. aureus* had a growth disadvantage, it is possible that fewer linezolid-resistant colonies would be selected by this approach. Thus, we may have underestimated the true incidence of linezolid resistance due to under-sampling. We also recently found that *S. aureus* is underdiagnosed in this population due to culture conditions (58). Because the patients studied were treated with a wide range of antibiotics, it is possible that some bacterial mutations are adaptive for antibiotics other than linezolid but confer cross-resistance. Finally, the novel mutations that we reported are associated with linezolid resistance, but we have not provided causal evidence of linezolid resistance by analyzing isogenic strains with and without each mutation individually.

### Conclusions

Linezolid is frequently used for patients with CF who have MRSA infections and worsening lung disease. Despite the repeated use of linezolid for some MRSA infections, relatively few subjects developed linezolid-resistant MRSA within this center. Persistence of linezolid-resistant *S. aureus* within these subjects was uncommon, possibly due to growth defects in linezolid-resistant strains. 23S rRNA mutations were the most common cause of linezolid-resistant MRSA. Hypermutating strains of MRSA could develop linezolid resistance through a combination of mutations affecting the 50S ribosome. As more molecular diagnostic methods are used in CF, these observations may aid in the earlier detection of antimicrobial resistance in patients requiring chronic antibiotic treatments.

## Data Availability

Genome sequencing data have been deposited in GenBank under the umbrella of BioProject PRJNA842383. Accession numbers for each isolate are provided in Fig. S1. Unassembled sequences are deposited in the sequence read archive. Bacterial strains are available to qualified researchers following the completion of a materials transfer agreement.

## SUPPLEMENTAL MATERIAL

Supplemental material spectrum.02084-23-s0001.docx Supplemental figures and tables.

ASM does not own the copyrights to Supplemental Material that may be linked to, or accessed through, an article. The authors have granted ASM a non-exclusive, world-wide license to publish the Supplemental Material files. Please contact the corresponding author directly for reuse.
