# Supplementary material for: Parallel evolution of linezolid-resistant Staphylococcus aureus in patients with cystic fibrosis
Source: Microbiol Spectr. 2023 Sep 19;11(5):e02084-23. doi: 10.1128/spectrum.02084-23 (PMC10581212; doi:10.1128/spectrum.02084-23)
Supplement: Supplemental material — Supplemental figures and tables. [file spectrum.02084-23-s0001.docx]

**Data Supplement for**

**Parallel Evolution of Linezolid Resistant *Staphylococcus aureus* in
Patients with Cystic Fibrosis**

By

Nicholas J. Pitcher, Andries Feder, Nicholas Bolden, Christian F. Zirbes, Anthony J. Pamatmat,
Linda Boyken, Jared J. Hill, Alyssa R. Bartels, Andrew L. Thurman,

Valerie C. Reeb, Harry S. Porterfield, Ahmed M. Moustafa, Paul J. Planet, and Anthony J. Fischer

**Supplemental Data 1.** GenBank Accession numbers for isolates analyzed in this study.

| **Isolate** | **Biosample Accession Number*** | **Genome Accession Numbers** | | **Short read sequencing data** | **Long read sequencing data** |
| --- | --- | --- | --- | --- | --- |
|  |  | **Short Read Assembly** | **Hybrid Assembly** |  |  |
| AF4001 | SAMN19677327 | JAHMTO000000000 |  | SRR25147157 |  |
|  | SAMN32369491 |  | CP115472 |  | SRR25147131 |
| HP20814.006 | SAMN15306981 | JACOAL000000000 |  | SRR25147156 |  |
|  | SAMN32369494 |  | CP115476 |  | SRR25147128 |
| AF2324 | SAMN19288831 | JAHNJU000000000 |  | SRR25147145 |  |
| HP20814.043 | SAMN15520220 | JACODR000000000 |  | SRR25147134 |  |
|  | SAMN32369495 |  | CP115477 |  | SRR25147127 |
| HP20814.051 | SAMN15520226 | JACODX000000000 |  | SRR25147125 |  |
| AF4003 | SAMN19677329 | JAHMTQ000000000 |  | SRR25147124 |  |
| HP20814.091 | SAMN15520227 | JACODY000000000 |  | SRR25147123 |  |
| AF2323 | SAMN18038513 | JAHNJV000000000 |  | SRR25147122 |  |
| AF2325 | SAMN19288832 | JAHNJT000000000 |  | SRR25147121 |  |
| AF4005 | SAMN19677331 | JAHMTS000000000 |  | SRR25147120 |  |
|  | SAMN32369493 |  | CP115474-CP115475 |  | SRR25147129 |
| HP20814.058 | SAMN15307023 | JACOCB000000000 |  | SRR25147155 |  |
| AF2246 | SAMN18038240 | JAHNMI000000000 |  | SRR25147154 |  |
| AF2247 | SAMN18038241 | JAHNMH000000000 |  | SRR25147153 |  |
| AF2248 | SAMN18038242 | JAHNMG000000000 |  | SRR25147152 |  |
| AF4006 | SAMN19677332 | JAHMTT000000000 |  | SRR25147151 |  |
| AF4008 | SAMN19677334 | JAHMTV000000000 |  | SRR25147150 |  |
| AF4009 | SAMN19677335 | JAHMTW000000000 |  | SRR25147149 |  |
| AF4010 | SAMN19677336 | JAHMTX000000000 |  | SRR25147148 |  |
| AF4011 | SAMN19677337 | JAHMTY000000000 |  | SRR25147147 |  |
| AF4012 | SAMN19677338 | JAHMTZ000000000 |  | SRR25147146 |  |
| AF4013 | SAMN19677339 | JAHMUA000000000 |  | SRR25147144 |  |
| AF4015 | SAMN19677341 | JAHMUC000000000 |  | SRR25147143 |  |
| AF4016 | SAMN19677342 | JAHMUD000000000 |  | SRR25147142 |  |
| AF4017 | SAMN19677343 | JAHMUE000000000 |  | SRR25147141 |  |
| AF4004 | SAMN19677330 | JAHMTR000000000 |  | SRR25147140 |  |
|  | SAMN32369492 |  | CP115473 |  | SRR25147130 |
| AF4007 | SAMN19677333 | JAHMTU000000000 |  | SRR25147139 |  |
| AF4014 | SAMN19677340 | JAHMUB000000000 |  | SRR25147138 |  |
| AF1552 | SAMN15306620 | JAIGBN000000000 |  | SRR25147137 |  |
| HP20814.062 | SAMN15307027 | JACOCF000000000 |  | SRR25147136 |  |
|  | SAMN32369496 |  | CP115478 |  | SRR25147126 |
| AF4002 | SAMN19677328 | JAHMTP000000000 |  | SRR25147135 |  |
| AF2001 | SAMN18038076 | JAHNUN000000000 |  | SRR25147133 |  |
| AF2002 | SAMN18038077 | JAHNUM000000000 |  | SRR25147132 |  |

*A second biosample was assigned when long read sequencing was performed. Isolates with long read sequencing are indicated in shaded cells.

**Supplemental Data 2. Prescription of Linezolid to people with CF.**

| **Year** | **Linezolid**  **Prescriptions or Orders, N** | **Patients prescribed linezolid, N** | **Patients with prescription data, N** |
| --- | --- | --- | --- |
| 2009* | 58 | 19 | 189 |
| 2010 | 59 | 25 | 208 |
| 2011 | 42 | 19 | 216 |
| 2012 | 54 | 24 | 233 |
| 2013 | 67 | 31 | 246 |
| 2014 | 73 | 36 | 245 |
| 2015 | 167 | 40 | 249 |
| 2016 | 127 | 45 | 254 |
| 2017 | 154 | 40 | 254 |
| 2018^†^ | 33 | 14 | 224 |
| All years | 834 | 111^‡^ | 346^‡^ |

* Earliest electronic prescription data was February 2009. ^†^ Incomplete year, last data from April 2018. ^‡^Some patients received prescriptions in multiple years.

**Supplemental Data 3.** Eligibility Criteria. N = 360 patients with CF have records between 2008 and 2018. Of these, N = 346 had complete records including both electronic prescriptions and clinical microbiology reports.

**Supplemental Figure 4.** Sequence of administration of antibiotics active against MRSA. Linezolid and tetracyclines were used after prior attempts at treatment with trimethoprim-sulfamethoxazole (TMP/SMX) and Vancomycin IV. There were significant differences in the order of treatment, Friedman’s rank sum test *P* < 0.001. Linezolid followed vancomycin IV (Wilcoxon signed rank test *P* = 0.01) and TMP/SMX (*P* < 0.001).

**Supplemental Figure 5. Chronicity of antibiotic exposure.** Each graph represents an electronic prescription timeline for patients receiving linezolid (N = 111, data in the four graphs at top) or for control subjects (N = 235, data in the four graphs at bottom). In each graph, subjects are represented on the vertical axis and dates on the horizontal axis. A date with a prescription is indicated by a small gray dot. Prescriptions for Linezolid, Trimethoprim/Sulfamethoxazole, Tetracyclines, or IV Vancomycin are indicated by a larger colored dot. The average number of prescriptions per patient is given at the top of the figure for each group. Subjects in both groups were treated repeatedly with antibiotics, but the number of subjects receiving treatment was higher in the linezolid group, as was the number of prescriptions per patient.

**Supplemental Data 6**. Number of unique dates with a linezolid order per patient.

**Supplemental Data 7.** Daily dose of linezolid prescribed for patients with cystic fibrosis. The typical dose was 1200 mg, equal to the maximum recommended adult dose for linezolid. Data were extracted from N = 351 prescriptions that provided explicit instructions on dosing schedule.

**Supplemental Data 8.** Course duration of linezolid prescribed for patients with cystic fibrosis. The typical duration was 14 days, although some prescriptions were significantly longer. Data were extracted from N = 146 prescriptions that explicitly indicated the duration of therapy.

**Supplemental Data 9.** Maximum number of refills authorized per patient on linezolid prescriptions, N = 111 patients with CF. Of 834 linezolid prescriptions, 66 prescriptions authorized one or more refills to allow for chronic treatment. Each of the four subjects with linezolid resistant *S. aureus* was offered refills of linezolid, ranging from 3 refills (Subject 1) to 11 refills (Subject 4).

**Supplemental Figure 10.** Phylogenetic tree drawn as in Figure 2, except that it includes AF4014, an ST582 isolate. Linezolid resistance is indicated with a black square and methicillin resistance with a red triangle. Isolates from this study are colored by subject; taxa without added color represent closely related genomes downloaded from public databases. Subject 3 (green) has polyclonal *S. aureus*, including ST5, ST105, and ST582. Isolates that were examined with long read sequencing are indicated with an asterisk. The collection times in year and month are indicated for each isolate.

**Supplemental Figure 11.** Phylogenetic analysis of Linezolid resistant *S. aureus*, drawn without using branch lengths to clearly display tree topology. Linezolid resistant isolates (MIC > 4) are indicated by a black square, MRSA are indicated by a red triangle. Isolates analyzed this study (N = 32) are color-coded by study subject. Non-colored branches (N = 36) represent genomes identified from GenBank that were among the top 5 most closely related genomes to those in our study. The tree is a maximum likelihood phylogenetic tree, with numbers underneath each branch of the tree indicating the confidence level of the node based on 100 non-parametric bootstrap pseudoreplicates. Separation of isolates that are shaded with the same color indicates polyclonality of strains cultured from the patient. For example, subject 3 (green) possesses at least three distinct strains. Strain sharing is evident between subject 3 and subject 2 (lavender); the linezolid resistant isolate AF4005 from subject 2 is most closely related to AF4007 from subject 3. All linezolid resistant isolates in this study emerged from ST5 or ST105 MRSA lineages. Within subject 1 (sky blue), phylogenetic analysis suggests linezolid resistance evolved twice, as AF2324 appears separate from the subject’s five other linezolid resistant isolates. Isolates that were examined with long read sequencing are indicated with an asterisk. The collection times in year and month are indicated for each isolate.

**Supplemental Data 12.** SNP distance between core genomes for isolates from Subject 1 (highlighted in gray) and the three nearest genomic references downloaded from NCBI. Several distances within this strain exceed an arbitrary limit of 60 SNPs. Darker green shading indicates greater proximity between isolates. The pairwise distance within the strain ranged from 24-204 SNPs. The average intrastrain SNP distance (from all pairwise distances enclosed in the triangle) was 126 SNPs. The average pairwise distance between members of the strain and the closest genomic reference was 169.

**Supplemental Data 13.** SNP distance between isolates from Subject 2 (light gray shade), the isolate AF4007 from Subject 3 belonging to the same strain (darker shade), and the nearest genomic references (white). Cells with darker green shading indicate greater proximity between isolates. The pairwise distance within the strain ranged from 4-193 SNPs. The average intrastrain SNP distance (from all pairwise distances enclosed in the triangle) was 108 SNPs. The average pairwise distance between members of the strain and the closest genomic reference was 143. Although AF4005 and AF4007 are > 60 SNPs apart from other isolates, we cannot disprove that they belong to the same strain by Clade Breaker analysis.

**Supplemental Data 14.** SNP distance between isolates from Subject 3. Isolates that did not belong to the dominant strain are highlighted in dark gray, including AF4014 (an ST582 isolate) and AF4005 (a distinct strain of ST105 with strong similarity to isolates from Subject 2). The intrastrain average indicates the average pairwise distance between isolates within the triangle. Darker green shading indicates greater proximity between isolates. The average distance between the three nearest genomes downloaded from NCBI and members of the strain (labels shaded in light gray) is indicated on the bottom at right. The average pairwise distance between members of the strain and the closest genomic reference was 124.

**Supplemental Data 15.** SNP distance between isolates from Subject 4. Darker green shading indicates greater proximity between isolates. AF2002 is the most distant, but we cannot disprove that it belongs to the same strain as the other isolates by Clade Breaker analysis. The average pairwise SNP distance between isolates that belong to the strain (enclosed within the triangle) was 98 and ranged from 2-230. The average distance between members of the strain and the nearest available genome downloaded from NCBI was 160.

**Supplemental Data 16.** SNP distance between representative isolates from different strains originating in different subjects.

**Supplemental Data 17**. Repeated appearance of a known linezolid resistance mutation (23S rRNA G2576T) in unrelated isolates. Because of ambiguity in the numbering system for 23S rRNA genes (1), we used reference *Escherichia coli* strain ATCC 8739 (GenBank accession NC_010468) to indicate position 2576. AF2324 (Subject 1) contains 6 copies of 23S rRNA; 2 alleles were T, and the remaining reads were ambiguous (K indicates either G or T). AF4004 (Subject 3) contains 5 copies of 23S rRNA; three alleles are G2576T. HP20814-062 (Subject 4) contains 6 copies of 23S rRNA, 2 are G2576T.

1. Beukers AG, Hasman H, Hegstad K, van Hal SJ. 2018. Recommendations To Address the Difficulties Encountered When Determining Linezolid Resistance from Whole-Genome Sequencing Data. Antimicrob Agents Chemother 62.
